# Identification of Balanced and Unbalanced Complex Chromosomal Rearrangement Involving Chromosomes 1, 11, and 15

**DOI:** 10.7759/cureus.16166

**Published:** 2021-07-04

**Authors:** Farzane Vafaeie, Masoume Ale Rasoul, Maryam Rahnama, Majid Mojarrad

**Affiliations:** 1 Medical Genetics Laboratory, Genetic Foundation of Khorasan Razavi, Mashhad, IRN; 2 Department of Medical Genetics, Mashhad University of Medical Sciences, Mashhad, IRN; 3 Genetic Research Center, Faculty of Medicine, Mashhad University of Medical Sciences, Mashhad, IRN

**Keywords:** complex chromosomal rearrangements, partial trisomy, karyotype analysis, recurrent abortion, g banding analysis

## Abstract

Chromosomal abnormalities are the common genetic factors that significantly impact fertility, miscarriage possibility and abnormal offspring with unbalanced karyotype. Complex chromosomal rearrangements (CCRs) refer to structural rearrangements which involve more than two breakpoints and often more than two chromosomes. According to the mode of transmission, they can be either familial or *de novo* rearrangements.

Here we report a complex chromosomal rearrangement leading to intellectual disability, speech delay and multiple dysmorphic features, including cleft lip and inguinal hernia. Proband karyotype shows 46,XY,ins (1::11) (q42→qter::q25) compatible to partial trisomy 1 q42→qter, while the karyotype of his mother was 45,XX, ins (1::15) (q42;q11.1→qter), t (1;11)(q42,q25) compatible to apparently normal female phenotype.

## Introduction

Complex chromosomal rearrangements (CCRs) refer to structural rearrangements which include more than two non-homologous chromosomes [1]. There are various classifications of CCRs based on the number of chromosomal breaks, the mode of transmission, balanced or unbalanced translocation, and the way of exchanging between three chromosomes to highly complex translocation [2].

Apparently, balanced CCRs carriers have a normal phenotype but the number of chromosomes and the number of breakpoints involved can vary greatly giving a variety of possible gametes. CCRs carriers are at a dramatically higher risk for infertility, recurrent abortions and abnormal offspring in comparison with classic translocations due to either malsegregation or recombination [3]. Most of familial CCRs are transmitted predominately through female carriers of balanced CCRs. In contrast, males balanced CCRs are often subfertile or sterile due to their chromosomes behavior during spermatogenesis [4].

Pedigree study and genetic testing, including karyotype analysis, are essential for genetic counseling of CCRs carriers [5]. Karyotype analysis is known as the gold standard method for prenatal cytogenetic diagnosis, including numerical and structural chromosomal aberrations. Partial aneuploidies in the offspring due to parental balanced chromosomal rearrangements can result in multiple congenital anomalies, intellectual disability, and other numerous dysmorphic features related to the size and origin of the involved segment [6]. Here, we report a case of 1q partial trisomy inherited from a carrier of CCR involving chromosomes 1, 11 and 15. Trisomy of the long arm of chromosome 1 is a rare chromosomal abnormality resulting from a parental balanced translocation. The clinical phenotypes of partial trisomy 1q syndrome are extremely diverse based on the location of breakpoints on chromosome 1. However, the major symptoms include short stature, multiple minor anomalies, and intellectual disability [7, 8].

## Case presentation

**Figure 1:**
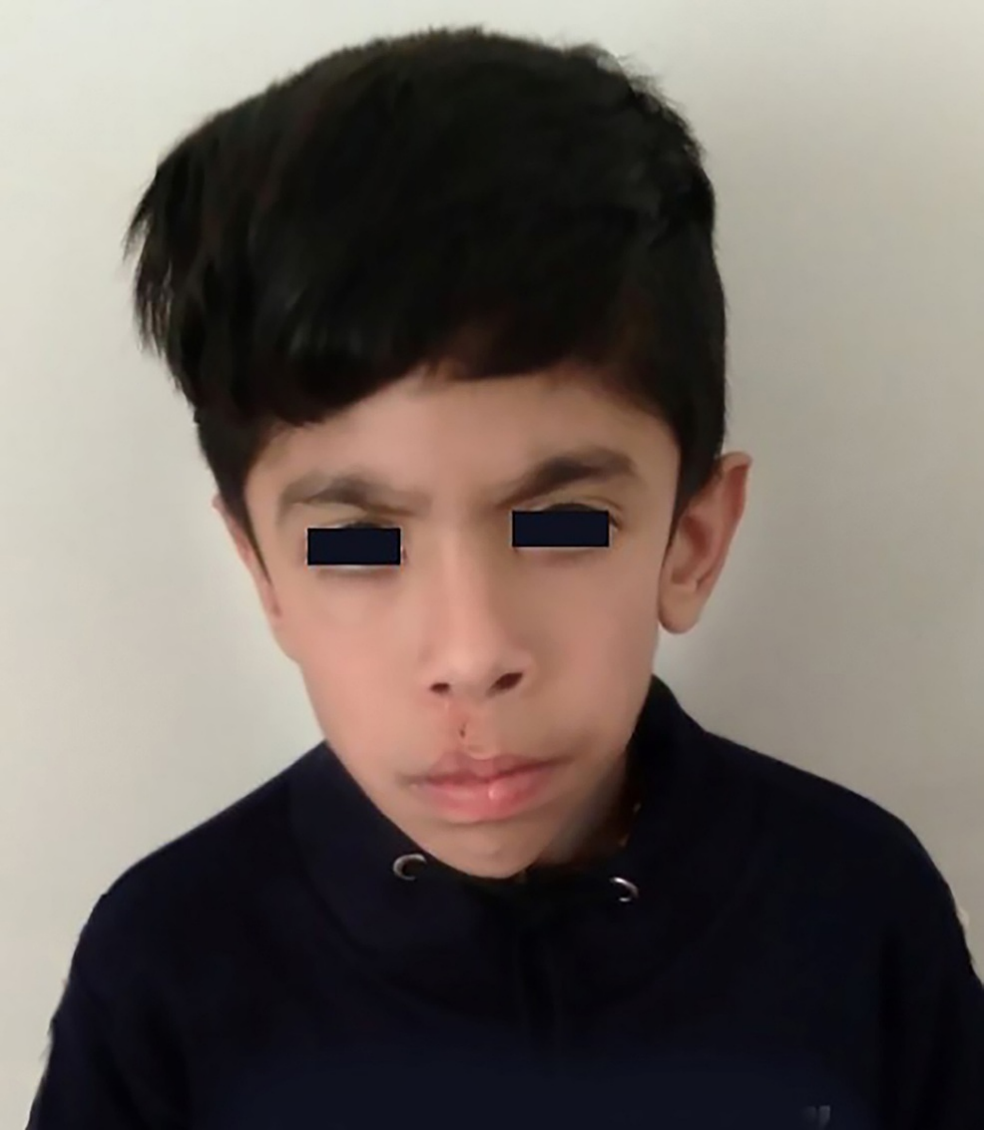
The proband image at the age of 7, showing facial dysmorphisms including cleft lip and long face with a prominent forehead and triangular face.

Our patient was 7-year-old boy with multiple congenital anomaly and intellectual disability. He was the first child of a non-consanguineous couple. He was born at 33 weeks of gestation with a birth weight was 2100 g. His length was 45 cm and head circumference was 38 cm. Delivery was performed by cesarean section because of fetal arrhythmia. The infant could not suck and was fed with breast milk through a gastric tube for one week. The patient was referred to a genetic counseling center with a clinical history of intellectual disability and speech delay. In a primary clinical evaluation, he had multiple dysmorphic features including cleft lip (repaired by surgery), inguinal hernia, macrocephaly and a long face with a prominent forehead (Figure 1). The proband's mother had one pregnancy loss before 20th weeks of gestation and infertility for five years. There was no family history of miscarriages or genetic disorders. Her age at menarche was 14 years, and she had regular menstrual cycles. Ultrasound imaging revealed a normal uterus and ovaries. According to patient clinical manifestations and presence of infertility and abortion, cytogenetic analysis was performed. 

**Figure 2:**
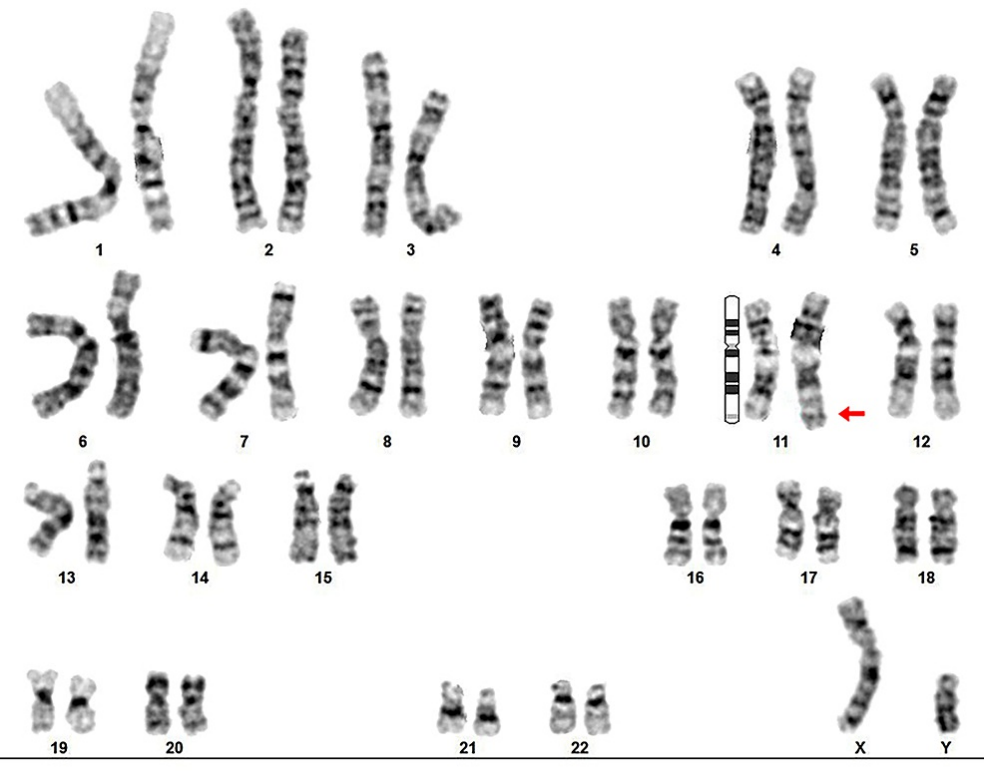
Chromosomal analysis of proband with familial insertion inherited from the mother. G-banding method at 650 bands per haploid on 50 spreads was performed.

**Figure 3:**
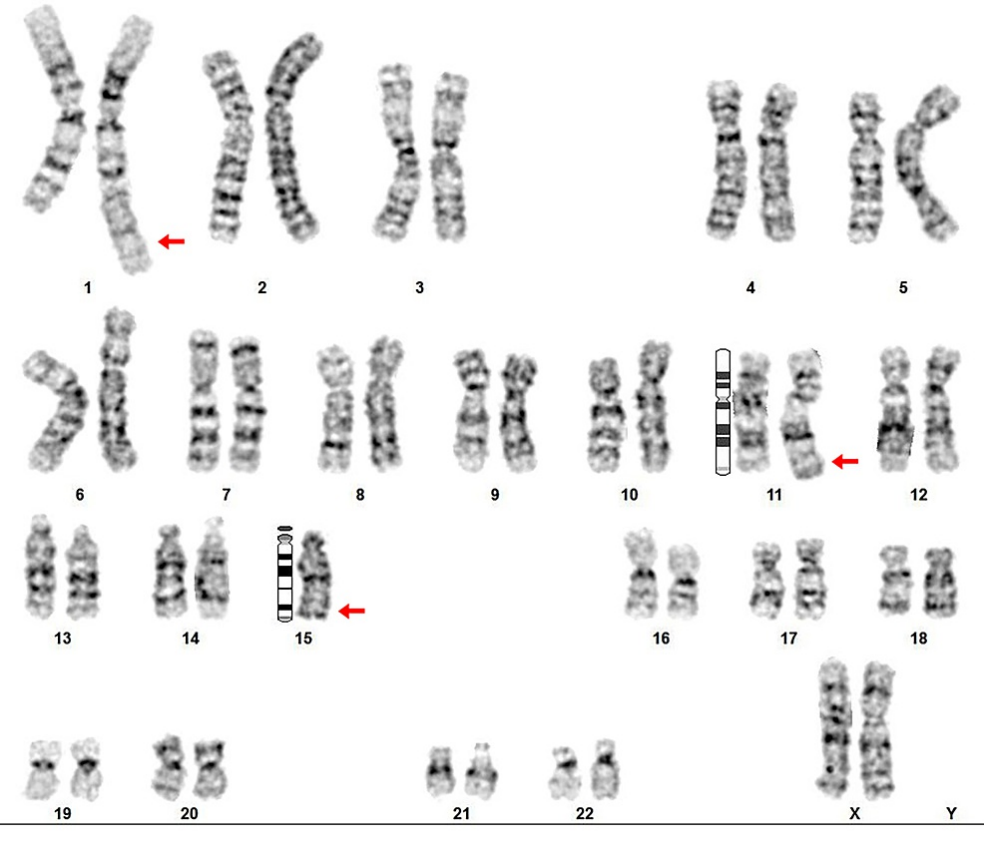
Complex chromosomal rearrangement in proband’s mother. G-banding method at 750 bands per haploid on 50 spreads was performed.

After taking informed consent from parents, peripheral blood sample from the proband and his parents was collected. Blood cells were cultured using RPMI 1640. The blood cultures were incubated at 37ºC for 4 days, the cell divisions were arrested in metaphases by adding colchicine 40 µM final concentration for 30 minutes before harvesting the lymphocytes. RBC were lysed using hypotonic 0.075 M KCl solution treatment for 30 minutes. The samples were fixed in a solution of 3:1 methanol-acetic acid and spread in microscopic slides. Karyotype analysis in a light microscope was carried out on G-banded chromosomes. Cytogenetic analysis of the patient's prometaphase chromosomes was done by using GenAsis software. Karyotype analysis of the proband showed a structurally abnormal unbalanced karyotype. An extra unknown chromosomal fragment was detected on chromosome 11q (Figure 2). The proband's father's karyotype was normal but the karyotype analysis of the mother revealed apparently balanced translocation involving the long arm of chromosome 1, the long arm of chromosome 11 and whole chromosome 15 at breakpoints 1q42, 11q22, and 15 q11 respectively (Figure 3).

As shown in figure 3, the most probable karyotype result of the mother could be 45, XX, ins (1::15) (q42;q11.1→qter), t (1;11)(q42,q25). According to chromosomal analysis of the mother, the proband inherited insertional fragment on chromosome number 11. Therefore, the proband's karyotype was 46, XY, ins (1::11) (q42→qter::q25) compatible to partial trisomy 1 q42→qter. According to normal G-banding chromosomal analysis of the maternal proband’s family, the mother’s karyotype was interpreted as a* de novo* balanced translocation.

## Discussion

**Figure 4:**
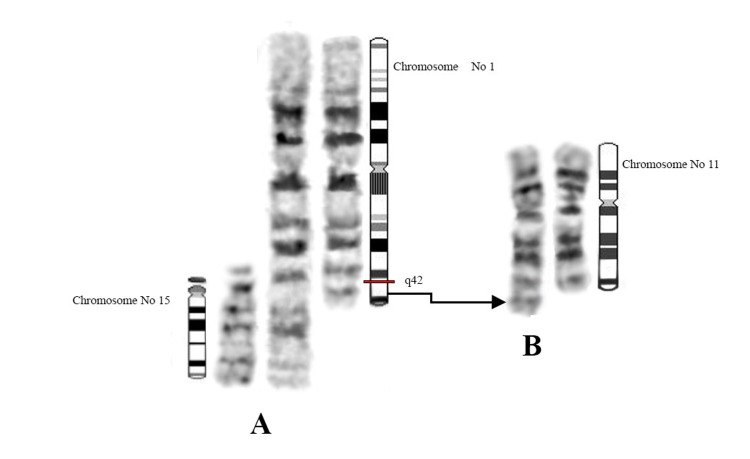
The translocations among three chromosomes in the mother of the proband.

**Figure 5:**
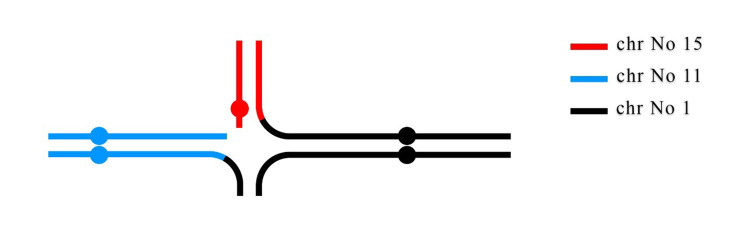
Theoretical pentavalent pachytene configuration adopted at meiosis I in the mother.

CCRs are structurally balanced or unbalanced aberrations involving more than two breakpoints on two or more chromosomes [9]. Chromosome rearrangements are more tolerated in female meiosis than male meiosis. Hence, approximately 75% of CCRs appear to be inherited maternally or are due to *de novo* rearrangements [10]. In the present case, karyotype analysis of the proband's parents revealed that CCRs originated maternally. The proband's mother, to our knowledge, is the first reported case of CCR with involvement of chromosomes 1, 11, 15. The total number of breaks in this rearrangement were three involving non-reciprocal translocation containing the insertion of the 15q11qter region to 1q42 and 1q42qter region to 11q22 (Figure 4). The mother's translocation was *de novo* and no familial history of chromosomal abnormality were observed. This karyotype explained presence of infertility problems and abnormal offspring in this family. The mother’s karyotype showed that derivative chromosomes could form a pentavalent structure in pachytene stage during meiosis I. The result of meiotic segregation in derivative chromosomes strongly depends on chromosome’s feature in pentavalent configuration. The pentavalent and the relative synaptic regions are shown in a diagram (Figure 5). For present CCRs, the theoretical modes of meiotic segregation are 2:3 (the only mode of segregation can result in normal/balanced gametes), 4:1 and 5:0 that leading to produce 32 different gametes. The possibility of normal gametes or balanced translocation is very low. A theoretical prediction of chromosomal segregation in gametes expected that only 6.25% of gametes would be normal or balanced translocation. Thus, early pregnancy loss, infertility and abnormal offspring are very likely.

**Figure 6:**
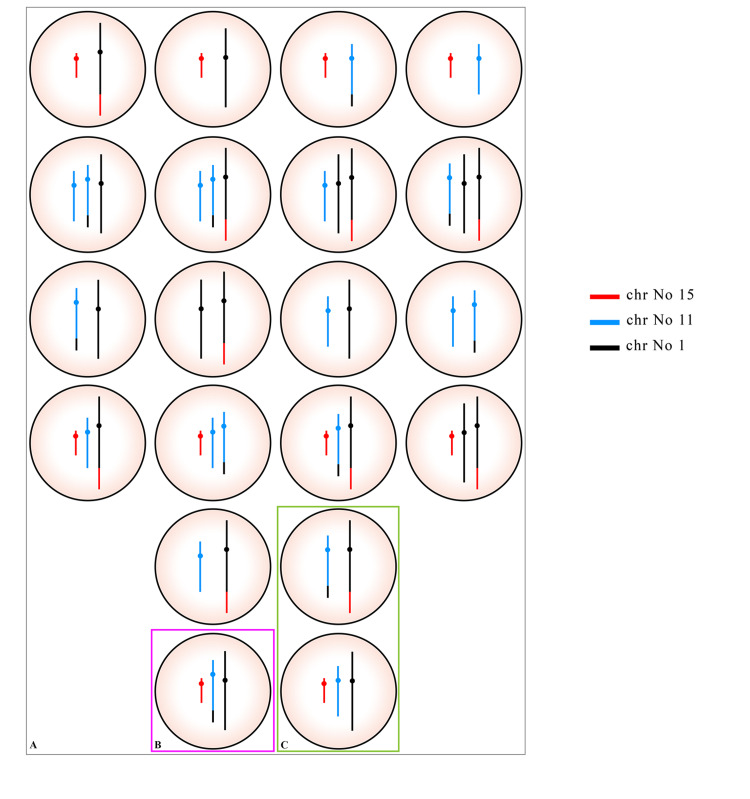
(A) Possible karyotypes of oocytes in mother through 2:3 segregation. (B) Segregation mode of the proband haploid. (C) Balanced haploid or normal complement.

Figure 6 shows all possible gametes in 2:3 mode of segregation. Normal or balance translocated gametes and proband haploid with an extra segment on chromosome 11 are generated by 2:3 segregation.

Proband was detected with partial trisomy 1q which is a relatively common form of heredity autosome trisomy [11-13]. Partial 1q trisomy syndrome is considered a rare disorder which can be classified according to breakpoint position as 1q32→qter or 1q42→qter. According to literature, partial trisomy of 1q without any other involving chromosomes often led to relatively mild phenotype which include macrocephaly, intellectual disability, developmental delay, triangular face and prominent forehead [11, 14, 15]. Cleft lip [16, 17] and inguinal hernia [18, 19] are rare symptoms that have been presented in few cases. When comparing the proband's clinical features described here with those reported cases that involve pure partial trisomy 1q42→qter syndrome, phenotypic similarities observed. According to the minimum involvement of chromosome 11q, in only the distal telemetric region, it can be concluded that the proband was affected by pure partial trisomy 1q42→qter.

Linking chromosome segmented duplication abnormalities and clinical phenotypes is a challenging process due to presence of various modulation factors such as gene-dosage effect, gene-gene interactions, modified gene effect, chromosome position effect and epigenetic influences. Some genetic factors such as difference in the size of translocated segment, the number of involved chromosomes, underlying breakpoint and non-genetic factors including age, sex and environment make genetic counseling very difficult for families with CCR [7, 20].

## Conclusions

In summery we present a *de novo* case of CCR with history of infertility, recurrent abortion and abnormal offspring. According to high risk of chromosomal abnormality in produced gametes, IVF-PGD (in vitro fertilization pre-implantation genetic diagnosis) using Array-CGH technique can be the best choice for this case.
